# Spatial Variation of Phosphorous Retention Capacity in Subsurface Flow Constructed Wetlands: Effect of Wetland Type and Inflow Loading

**DOI:** 10.1371/journal.pone.0134010

**Published:** 2015-07-28

**Authors:** Guangwei Yu, Meijuan Tan, Yunxiao Chong, Xinxian Long

**Affiliations:** Department of Environmental Science and Engineering, College of Natural Resource and Environment, South China Agricultural University, Guangzhou, 510642, China; Shandong University, CHINA

## Abstract

For verification of spatial distribution of phosphorous retention capacity in constructed wetlands systems(CWs), two horizontal subsurface flow(HSSF) CWs and two vertical subsurface flow(VSSF) CWs, using sand as substrate and *Typha latifolia* as wetland plants, were constructed and put into use for synthetic wastewater treatment. Five months later, significant spatial variations of TP and inorganic phosphorus(Ca-P, Fe-P and Al-P) were observed, which were found to be greatly affected by CWs type and hydraulic loading. The results revealed that though spatial distribution of Fe-P and Al-P displayed a similar order of substrate content as "rhizosphere" > "near-rhizosphere" > "non-rhizosphere" and "inflow section" > "outflow section" regardless of types and loading, the distribution of Ca-P was positively correlated to that of Fe-P and Al-P in HSSF CWs, while negative correlation was shown in VSSF CWs. As a result, TP spatial distribution in HSSF CWs demonstrated a greater dissimilarity than that in VSSF CWs. For HSSF CWs with low hydraulic loading, the lowest TP content was found in non-rhizosphere substrate of outflow section, while the highest one was discovered in rhizonsphere substrate of inflow section. The values in 6 parts of areas ranged from 0.138 g·kg^-1^ to 2.710 g·kg^-1^, which also were from -33.5% to 1209% compared to the control value. On contrast, spatial difference of TP content in substrates of VSSF CWs was insignificant, with a variation ranging from 0.776 g·kg^-1^ to 1.080 g·kg^-1^, that was 275% to 421% higher than the control value. In addition, when hydraulic loading was increased, TP content in VSSF CWs sharply decreased, ranging from 0.210 g·kg^-1^ to 0.634 g·kg^-1^. Meanwhile, dissimilarity of TP spatial distribution in HSSF CWs was reduced, with TP content ranging from 0.258 g·kg^-1^ to 2.237 g·kg^-1^. The results suggested that P spatial distribution should be taken into account for CWs design and operation.

## Introduction

As low-cost approaches, constructed wetlands(CWs) were widely used for the treatment of municipal, industrial, aquacultural and agricultural wastewater, in which phosphorus was the main pollutant that constructed wetlands aimed at removing[1–4]. Unlike the nitrogen, which could be eliminated off the system by nitrification and denitrification, phosphorous in wastewater was usually removed by retaining in the constructed wetland system[5,6]. Mechanisms of phosphorus removal in constructed wetlands had been fully investigated and could be described as sorption, precipitation, biomass uptake, peat accretion and burial, and so on[6]. Current researches had demonstrated that sorption and precipitation by substrate were the major process for P removal in CWs since the capacities of other processes were limited. Therefore, P retention capacity, or the saturation potential of the substrate had been considered as a crucial parameter for substrate selection and longevity evaluation of the CWs[7,8].

Generally, the P-sorbing capaity was determined by the chemical and physical properties of substrates, for example, the specific surface area and the content of Ca, Al and Fe in substrates[9]. However, as a whole system, during the treatment of wastewater the P sorption process in CWs was also greatly affected by the environmental factors (e.g. pH, ORP and DO)[10,11] and the operation factors, e.g. temperature and inflow loading rate(or hydraulic retention time)[12]. Furthermore, these factors always show an interaction effect for each other. Take ORP for example, it was agreed that substrate could adsorb more P under aerobic condition than did under anaerobic condition[13–15].On the other hand, ORP would be greatly influenced by hydraulic design(type of CWs), mode of operation, plants and seasons[14,16]. Thus, all these factors would affect P-sorbing capacity directly or indirectly as well. In addition, pH was always reported to be another important parameter determining the adsorption and desorption process of P in CWs, which was also closely related to the variation of ORP and microbial activity when treated wastewater contained massive organic pollutants. For example, under anaerobic condition, hydrolytic acidification of organic matter could change the physico-chemical features of substrates, such as decrease of pH level[17], reduction of Fe(Ⅲ) to Fe^2+^ [13], extraction and release of Fe and Al [18]- and so on.

Therefore, it could be suggested that the P retention process would be totally different when these parameters(pH, ORP, inflow loading, et al.) altered, whether in different type of CWs or in different parts of a single CW system. In fact, researches had confirmed that during the treatment of wastewater, environmental factors were variable and could show a significant dissimilarity in different physical parts of a CWs[19–21]-. García et al, (2003)[19] found that in HSSF CWs, ORP spatially increased from the inlet to the outlet. Ding, et al, (2014) [21]observed that pH in upper layer was slightly lower than the one in bottom layer, while ORP and DO in the upper layer were higher than those in bottom layer in HSSF CWs. In addition, spatial distribution of microbial communities and activities had been reported in different CW types[22,23]. Therefore, it could be suggested that the P retention process would be totally different in various parts of CW system. However, to what extent the spatial variation of environmental factors in CWs would contribute to the spatial variation of P retention capacity of substrate, was lack of understanding currently.

As mentioned above, P retention capacity had been treated as a parameter for the CWs longevity assessment[7, 24]. If there is significant P retention variation existing within the CW system, the longevity of substrate may be overestimated or underestimated due to the inhomogeneous distribution of P accumulation. Additionally, it is more effective and economical to construct a wetland system with different substrates according to the spatial distribution of P retention capacity to avoid partial saturation, especially in large scale of practical operation. Thus, it is imperative to assess the spatial variation of P retention capacity in substrate during the operation of wastewater treatment.

However, recent researches on the variance of P retention capacity mostly focused on the effect of substrates, types, inflow loadings, wetland plants, seasons and so on, which always took the whole CW systems as a study subject[24,15,25]. Few of them emphasized on the spatial distribution of P retention capacity within a single system, not to mention dividing its substrate zones into rhizosphere, near-rhizosphere and non-rhizosphere. Actually, in subsurface flow CWs, oxygen from plant root was the key source for oxygen transfer inside CWs, thus the redox condition could vary in parts different from the rhizosphere[26].

Our previous research[27] had found out that distribution of P retention in CWs with wetland plants was different in rhizosphere from that in non-rhizosphere areas compared to the system without plants. However, for the complete understanding of the spatial distribution of P retention in substrate of CWs, affecting factors like type and inflow hydraulic loading needed to be taken into account. Therefore, The purpose of this work was to verify the spatial variation of TP and inorganic P(Ca-P, Fe-P and Al-P) retention capacity in HSSF CWs and VSSF CWs under high and low hydraulic loading.

## Materials and Methods

### Construction of Constructed Wetlands System

**Fig 1:**
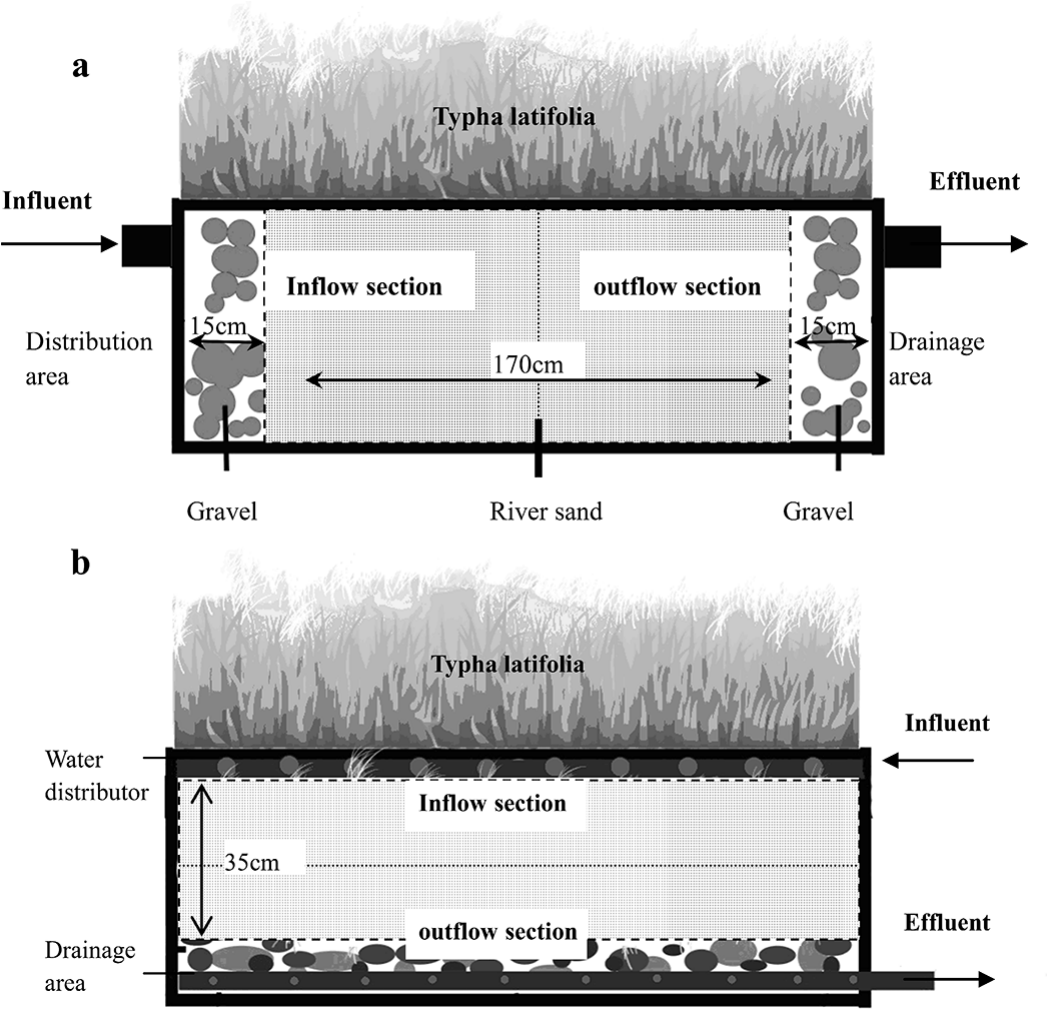
Construction of CWS with horizontal subsurface flow(a) and Vertical subsurface flow(b).

Four constructed wetland systems (CWS), two with horizontal subsurface flow (HSSF) and two with vertical subsurface flow(VSSF), were designed and constructed in laboratory. The framework of CWS was made of PVC with dimensions of 200cm in length, 100cm in width and 50cm in height. As showed in Fig 1A, the HSSF CWS was composed of inflow section and outflow section. The forepart of inflow section and the back part of outflow section (with length of 15cm) were designed as the wastewater distribution area and the drainage area, in which gravel media with a diameter of 3–5cm was filled in. The middle set of the systems (with length of 170cm) was designed as the main body of substrate, which was filled with common river sands with a sizes of 1–2mm as the filter media. In VSSF CWS, as seen in Fig 1B, wastewater drainage area was set at the bottom of the systems and filled with 15cm height of gravel media as well. In the upper part of the system, 35cm height of river sand was added as the main body of substrate. Similarly, the mainbody was artificially divided into inflow section and outflow section from the middle line. Chemical analysis of river sand in our previous research[27] showed that the main component of sand was SiO_2_, and the content of iron, aluminum and calcium, those elements that were related with P precipitation, were 9.10g/kg, 14.77 g/kg and 0.14 g/kg, respectively.

When the four CWs were built and the substrates were filled, 5 plants of *Typha latifolia* (one of the common species that widely used as wetland plants in south china) were planted uniformly on the main body of substrate in each CWs in early May 2013. For each system, 10L of nutrient solution (with 10 mg·L^-1^ of nitrogen and mg·L^-1^ of phosphorous) was added every 10 days. A month later, the number of *Typha latifolia* increased to 20 (with a density of 10 plants·m^-2^) and the plants fully covered the plate area of the CW systems. Then all the CW systems were put into use.

### Characteristic of Experiment Wastewater

Experiment wastewater used in this work was synthetic wastewater, consisted of glucose, phosphate, ammonium and trace elements. The characteristic of wastewater was measured and the results were described as follows.

Total Nitrogen(TN): 27.92–43.55 mg·L^-1^, Ammonium Nitrogen (NH_4_^+^-N): 27.59–36.66 mg·L^-1^, Total Phosphorous(TP):1.25–4.22 mg·L^-1^ and Chemical Oxygen Demand(COD): 113.5–262.83 mg·L^-1^.

### Operation Condition

The experiment was carried out from July 2013 to December 2013. Continuous flow mode was employed and both HSSF CWs and VSSF CWs were conducted under the high and low hydraulic loading conditions of 100×10^-3^m·d^-1^ and 60×10^-3^m·d^-1^.

### Sampling

#### Wastewater samples

Wastewater in the influent and effluent of the four CWs systems was sampled every 5 days, and TN, NH_4_^+^-N, TP and COD were measured respectively. Removal rate R of each index was calculated with the following formula:

$$\text{R}=\left({\text{c}}_{\text{in}}-{\text{c}}_{\text{out}}\right)/{\text{c}}_{\text{in}}\times 100\%$$

where C_in_ and C_out_ are the average values of each index in influent and effluent.

#### Substrate Samples

Before being filled into CWs, substrate sand was sampled for total phosphorus and inorganic phosphorus(Ca-P, Fe-P and Al-P) measurement, and the results were treated as control values. Then at the end of the experiment, substrate samples in different areas in four CWs were collected and measured again according to the following steps:

Firstly, the PVC framework of the CWs was disassembled, the residual water in CWSs was drained, and the aboveground of *Typha latifolia* was cut and removed, leaving about 3cm of stem with the underground part.

Then, substrate of CWs was zoned and samples were collected from different areas. For the horizontal subsurface flow CWs, distribution and drainage areas with gravel medium at both side of the systems were separated and removed firstly. Afterward, according to the space relationship of plant root and the substrate, the mainbody of substrate with river sand was divided into 6 areas as rhizosphere in inflow section, near-rhizosphere in inflow section, non-rhizosphere in inflow section, rhizosphere in outflow section, near-rhizosphere in outflow section and non-rhizosphere in outflow section. When being sampling, sandy substrate which could be naturally peeled off from the plant root by gravity was defined as non-rhizosphere substrate. Substrates which attached to plant root but could be shaken off from the root by artificial shaking (about 5 to 10 min until no substrate dropping down) was defined as near-rhizosphere substrate. And substrates those adhered to the roots tight that can not be shaken off was defined as rhizosphere substrate. In this work, rhizosphere substrate was scraped and collected from roots after two days of air drying. For vertical subsurface flow CWS, the mainbody of substrate was divided into 4 areas as rhizosphere substrate in inflow section, near-rhizosphere substrate in inflow section, non-rhizosphere substrate in inflow section and non-rhizosphere substrate in outflow section since there was no plant root in the area of outflow section. The mass percentage of different parts of substrates was shown in Table 1.

**Table 1:** Percentages of different parts of substrates(dry weight) in four CWs (%).

Hydraulic Load:		60×10^−3^ (m·d^-1^)		100×10^−3^ (m·d^-1^)	
Areas of substrates		Inflow	Outflow	Inflow	Outflow
HSSF	rhizosphere	0.05	0.11	0.03	0.09
	near-rhizosphere	0.11	0.71	0.22	0.54
	non-rhizosphere	99.84	99.19	99.75	99.37
VSSF	rhizosphere	0.01		0.05	
	near-rhizosphere	0.66		0.65	
	non-rhizosphere	40.64	58.69	37.71	61.59

Finally, substrates in different zone of all CWS were sampled for the test of TP and inorganic phosphorus according to the "sample quartering method" for soil [28]. For each substrate parts, triple samples were taken for measurement. Here, the inorganic phosphorus in substrate was classified into 3 types as Ca-bound phosphorus (Ca-P), Fe-bound phosphorus(Fe-P) and Al-bound phosphorus(Al-P).

### Measurement and Data Analysis

TN, TP, NH_4_^+^-N and COD concentration in influent and effluent wastewater were measured according to national standard methods for surface water and wastewater monitoring[29]. TP in substrate was analyzed by Mo-Sb colorimetry methods after digestion with HClO_4_-H_2_SO_4_ according to standard methods for soil analysis[28]. The classification and determination of Al-P, Fe-P and Ca-P in substrate were conducted by the standard methods for measurement of inorganic phosphorous in acidic and neutral soil[28].

SPSS 16.0 and SigmaPlot 10.0 software were used for the ANOVA analysis and drawing.

## Results and Discussion

### Treatment Efficiency of Four CW Systems

Generally, high phosphorous removal rate would be achieved in new CWs due to the high phosphorous adsorption capacity of new substrate. As shown in Table 2, the removal rates of TP in vertical subsurface flow CW systems were over 80%, while the values of horizontal subsurface flow systems were over 70%, which indicated that most amount of phosphorous in wastewater was retained in the CW systems, and the P retention capacity of vertical subsurface flow CW systems was a little higher than that of horizontal subsurface flow systems.

**Table 2:** Removal rate of main pollutants in constructed wetlands.

Types	Hydraulic Load(m·d^-1^)	TN(%)	NH_4_^+^-N(%)	COD(%)	TP(%)
HSSF	60×10^−3^	24.81±16	44.74±39	76.00±28	72.84±20
	100×10^−3^	24.91±22	19.51±21	63.91±36	74.13±25
VSSF	60×10^−3^	30.80±27	75.71±13	82.89±16	84.76±12
	100×10^−3^	34.64±25	66.61±26	79.41±20	81.30±17

Table 2 also revealed that high COD treatment efficiency was achieved in the four CW systems. It showed that vertical subsurface flow CW systems could get a higher COD removal rate than that done by horizontal subsurface flow CW systems. Moreover, higher ammonium removal rate was observed in vertical flow CW systems as well. These results were supported by relevant researches, which confirmed that BOD and ammonium removal rates were higher in vertical flow compared to those in horizontal subsurface flow[6,30].

On the other hand, removal rates of total nitrogen of the four CW systems were quite low(24%-35%). Denitrification process was usually considered to be limited under high oxygen concentration condition, resulting in high ammonium removal rate but low TN reduction in VSSF CWs[6]. On the contrary, though denitrification was suggested to be faster in horizontal subsurface flow than that in vertical flow, removal rate of ammonium and TN were both low in HSSF CWs in this work because of the restriction of nitrification process, as shown in Table 2.

### Spatial Variation of TP in Two Types of CWs

**Fig 2:**
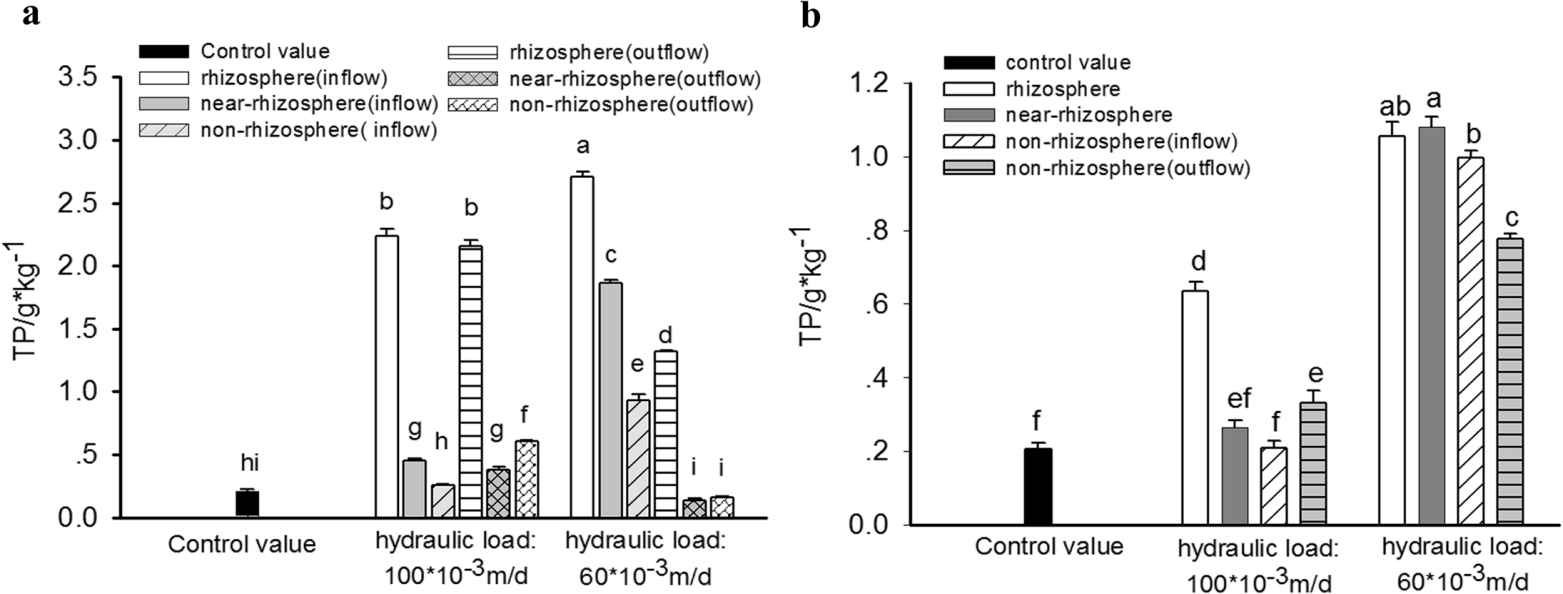
Content of TP in substrates in different areas of HSSF CWs(a) and VSSF CWS (b)with high and low hydraulic loading.

Spatial variations of TP retention in substrates of HSSF CWs and VSSF CWs after 5 months of wastewater treatment were shown in the Fig 2, which indicated that hydraulic characteristic(e.g. type and hydraulic loading) had significant influence on TP retention and spatial distribution in the CWs.

It was found that in low hydraulic loading systems, substrates in HSSF CWs showed a more remarkable difference in TP spatial distribution than that in VSSF CWs. In HSSF CWs, TP content in inflow section (front part) was obviously higher than that in the outflow section(back part). TP content in rhizosphere, near-rhizosphere and non-rhizosphere substrates in inflow section were 2.710 g·kg^-1^, 1.864 g·kg^-1^ and 0.933 mg·kg^-1^, respectively, increased by 1209%, 800% and 351% compared to the control value(0.207 mg·kg^-1^). It also showed a significant spatial TP gradient from rhizosphere to near-rhizosphere to non-rhizosphere. Yet, TP content in rhizosphere substrate in outflow section was 1.316 g·kg^-1^, only 535% higher than that of control value, and 51.4% less than the value in inflow section. Furthermore, TP content in near-rhizosphere and non-rhizosphere substrates were 0.138 g·kg^-1^ and 0.159 g·kg^-1^, 33.5% and 23.4% lower than the control value, indicating that TP release was occurred in these areas. On contrast, though similar result of "inflow section> outflow section" was found in VSSF CWs with low hydraulic loading, the vertical variation of TP retention presented in VSSF CWs was much less than that in HSSF CWs. TP content in non-rhizoshpere substrate in outflow section(bottom layer) was 0.776 g·kg^-1^, increased by 275% compared to the control value, which was only 22.2% lower than the value in non-rhizoshpere substrate in inflow section(upper layer). Fig 2B showed that TP content in rhizosphere, near-rhizhosphere and non-rhizosphere substrate in inflow section of VSSF CWs were 1.055 g·kg^-1^, 1.080 g·kg^-1^ and 0.997 g·kg^-1^, respectively, increased by 410%, 421% and 382% compared to the control value, indicating spatial TP gradient in this section was unconspicuous. In addition, though TP content in rhizosphere and near-rhizosphere substrates in HSSF CWs were much higher than the values in VSSF CWs, the higher TP content in non- rhizosphere substrate in VSSF CWs than that in HSSF CWs meant that VSSF CWs could retain more TP than that done by HSSF CWs since non-rhizosphere substrate occupied the major amount of total substrates in CWs.

Furthermore, Fig 2 also revealed that TP retention in two types of CWs was greatly affected by variation of hydraulic loading. As shown in Fig 2B, increase of hydraulic loading could significantly decrease the total TP retention of VSSF CWs. When inflow loading was increased from 0.06m/d to 0.1m/d, TP content in substrates in rhizosphere, near-rhizosphere, non-rhizosphere in inflow section and non-rhizosphere in outflow section sharply decreased to 0.634 g·kg^-1^, 0.265 g·kg^-1^, 0.210 g·kg^-1^ and 0.331 g·kg^-1^, respectively, which were 39.9%, 75.5%, 79.0% and 57.3% lower than values in low loading system. It seemed that TP retention capacity was obviously limited in all areas of substrates in VSSF CWs under high hydraulic loading condition. In contrast, when hydraulic loading of HSSF CWs was increased(Fig 2A), though TP content in rhizosphere, near-rhizosphere and non-rhizosphere substrates in inflow section decreased by 17.5%, 75.8% and 72.4% compared to values in low loading system, the values in outflow section increased by 63.7%, 172.4% and 281.2% compared to low loading values. As a result, the variation of TP spatial distribution between the inflow section and outflow section was reduced, but no significant influence on total TP retention of the whole system was observed.

### Spatial Variation of Inorganic Phosphorus in Substrate

Percentage of inorganic phosphorus (Ca-P, Fe-P and Al-P) to TP in different areas of substrates in four CWs was calculated in this work, and it showed that Ca-P, Fe-P and Al-P in non-rhizosphere substrates occupied the major amount of the TP(60%-90%), indicating that phosphorus was removed mainly by adsorption and precipitation of substrates.

**Fig 3:**
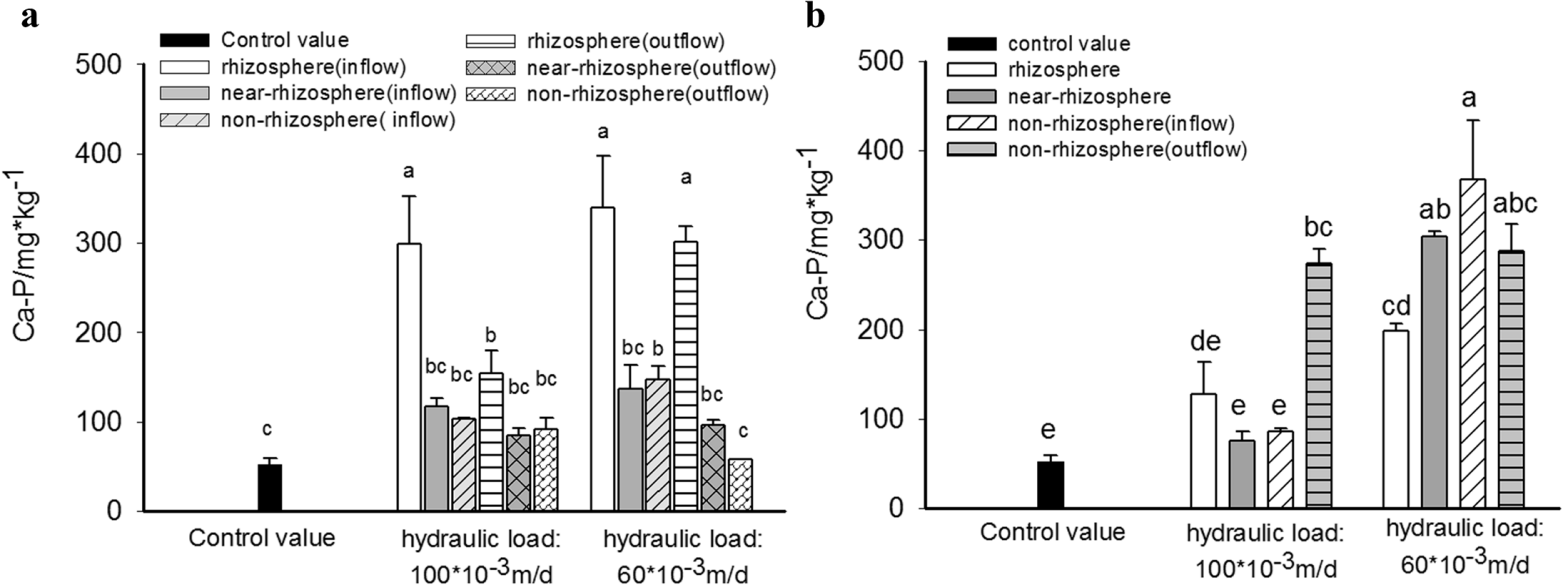
Content of Ca-P in substrates in different zone of HSSF CWS(a) and VSSF CWS(b) with high and low hydraulic loading rate.

Spatial variations of Ca-P retention in substrates of HSSF CWs and VSSF CWs were shown in the Fig 3. Among 6 sections of HSSF CWs, it was rhizosphere with substrate that contained the highest Ca-P content. Its Ca-P level was much higher than others and the control value in each HSSF CW. In low hydraulic loading system, Ca-P content in rhizosphere, near-rhizosphere and non-rhizosphere substrate increased by 544.0%, 159.5% and 179.2%(in inflow section) and 471.3%, 82.3%, and 9.8% (in outflow section) compared to the control value(52.7 mg·kg^-1^), respectively. In contract, Ca-P content in inflow section in VSSF CWs with low inflow loading was opposite to that in HSSF CWs, where the lowest Ca-P content was found in rhizosphere substrate, while the highest was discovered in non-rhizonsphere substrate. However, Ca-P contents in rhizosphere, near-rhizosphere, and non-rhizosphere in inflow section and outflow section were all significantly higher than the control value, which increased by 277.2%, 477.7%, 596.8% and 445.2%, respectively.

In addition, when hydraulic loading was increased, great influence was shown on the spatial distribution of Ca-P in VSSF CWs except for non-rhizosphere substrate in outflow section, in which Ca-P content was 419.2% higher than the control value. Ca-P content in other substrates all sharply decreased compared to those in low loading system. Ca-P content in rhizosphere, near-rhizosphere and non-rhizosphere in inflow section dropped by 35.9%, 75.3% and 76.6% compared to values in low loading system. In contrast, similar to the effect on TP distribution, differences of Ca-P distribution in inflow section and outflow section in HSSF CWs were reduced with the increase of inflow loading. Ca-P content decreased in non-rhizosphere substrate of inflow section, while increased in outflow section. However, Ca-P content in rhizosphere substrates was still much higher than that in other areas.

**Fig 4:**
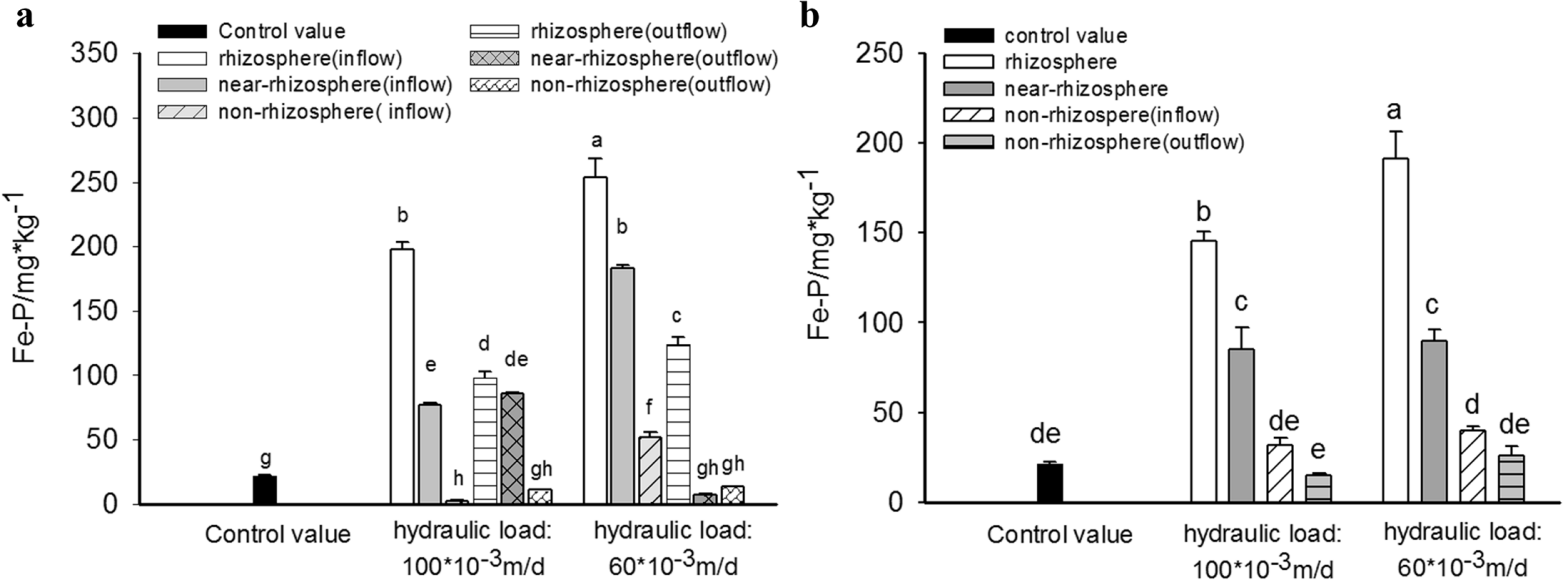
Content of Fe-P in substrates in different zone of HSSF CWS(a) and VSSF CWS(b) with high and low hydraulic loading rate.

Fig 4 revealed that spatial distribution of Fe-P in different substrates in HSSF CWs and VSSF CWs both showed a similar order as "rhizosphere" > "near-rhizosphere" > "non-rhizosphere" and "inflow section" > "outflow section".

As showed in Fig 4A, in inflow section of HSSF CWs with low hydraulic loading, Fe-P content in rhizosphere, near-rhizosphere and non-rhizosphere substrate were 1068.2%, 744.5% and 140.0% higher than the control value(21.7 mg·kg^-1^). However, in outflow section, only the Fe-P content in rhizosphere substrate increased by 471.9%, while in near-rhizosphere and non-rhizosphere substrate, it dropped down by 65.5% and 38.5% compared to the control value, which indicated that Fe-P desorption was occurred in these two areas. In contrast, when hydraulic loading was increased, Fe-P content in rhizonsphere, near-rhizonsphere and non-rhizosphere substrate in inflow section remarkably decreased. Specially, Fe-P content in non-rhizospere substrates in inflow section were 88.0% lower than the control value, which suggested that desorption of Fe-P in this area was intensified in high loading system. However, Fe-P content in near-rihzosphere substrate in outflow section increased by 1044.9% compared to values in low loading system, resulting in that the variation of Fe-P spatial distribution in inflow section and outflow section was reduced.

In addition, in VSSF CWs with low hydraulic loading (Fig 4B), Fe-P content in substrates in rhizosphere, near-rhizosphere, non-rhizosphere in inflow section and non-rhizosphere in outflow section were 781.0%,313.5%,84.8% and 20.3% higher than the control value. Nevertheless, in high loading system, Fe-P content in these four substrates decreased and were only 570.3%, 292.4%, 46.0% and -30.8% higher than the control value. The figure -30.8% meant that Fe-P desorption was occurred in non-rhizosphere substrate in outflow section due to the increase of inflow loading.

**Fig 5:**
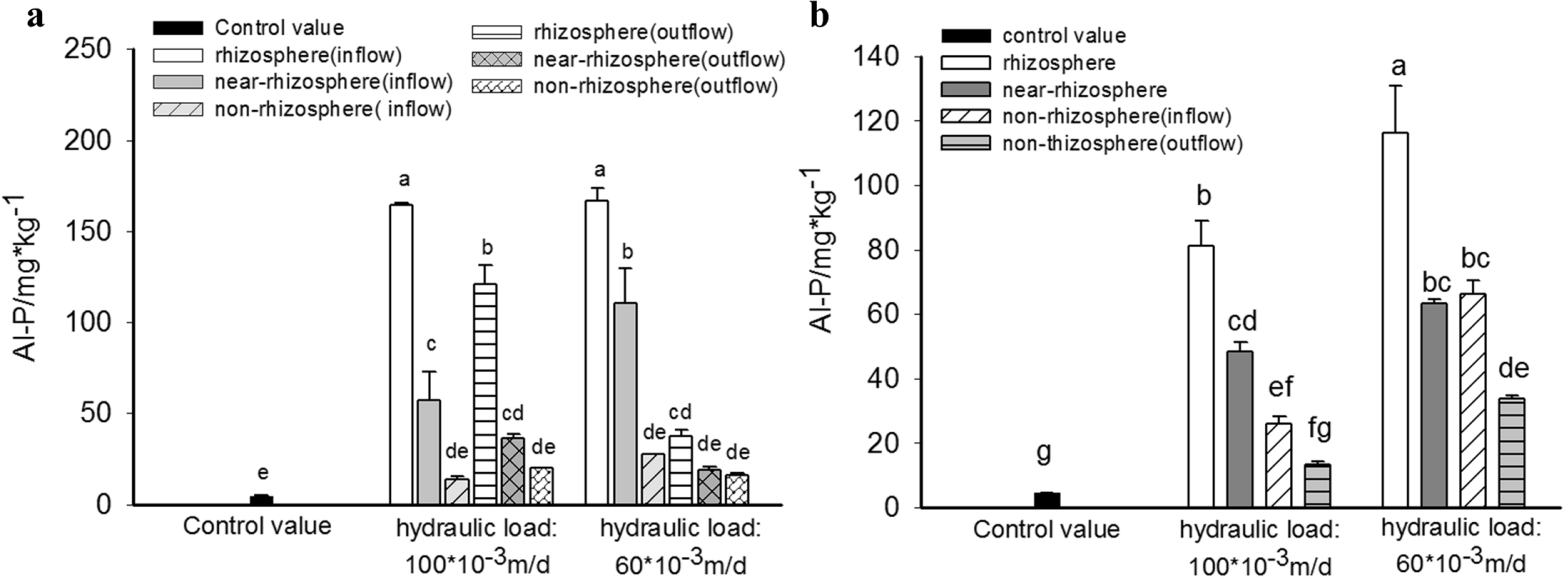
Content of Al-P in substrates in different zone of HSSF CWS(a) and VSSF CWS(b) with high and low hydraulic loading rate.

Spatial variation of Al-P retention in substrates of HSSF CWs and VSSF CWs were similar to spatial distribution of Fe-P, as shown in the Fig 5. In low hydraulic loading HSSF CWs(Fig 5A), Al-P content in rhizosphere, near-rhizosphere and non-rhizosphere substrate increased by 3624.5% 2372.3% and 515.0% (in inflow section) and 736.8%, 322.0% and 258.7%(in outflow section), respectively, compared to the control value(4.47 mg·kg^-1^). It indicated that Al-P content in substrates in inflow section was significant higher than that in outflow section, and in each section, Al-P content obeyed the order of "rhizosphere" > "near-rhizosphere" > "non-rhizosphere". In contrast, in high loading system, Al-P content decreased in inflow section while increased in outflow section. Due to Al-P content in rhizosphere substrate in outflow section increased by 223.0% compared to values in low loading system, variation of Al-P distribution in inflow section and outflow section was reduced as well.

For VSSF CWs system(Fig 5B), the increase of inflow loading sharply decreased the Al-P content in four kinds of substrates. Al-P content in substrates in rhizosphere, near-rhizosphere, non-rhizosphere in inflow section and non-rhizosphere in outflow section dropped down from 2497.1%, 1313.1%, 1381.9% and 655.1% higher than the control value to 1713.5%, 979.2%, 482.1% and 199.3% higher than the control value, when hydraulic loading was transferred from 0.06m/d to 0.1m/d.

## Discussion

### Effect of Type and Inflow Loading on Phosphorus Spatial Distribution

As mentioned above, affecting factors of P retention in substrates were complicated and always interactive. Generally, the spatial distribution of TP in CWs was closely related to spatial variation of pH and ORP level, which were greatly influenced by oxygen transfer, pollutant distribution and microbial activity within the system[14,23].

In subsurface flow constructed wetland systems, oxygen is usually supplied by wetland plants. It first releases into rhizosphere substrate and then transfers to near-rhizosphere and non-rhizosphere substrate, which serves as the reason why ORP always shows the same spatial distribution fate within CWs[26]. During this process, hydraulic characteristic (type and hydraulic loading) was found to be of great importance on oxygen retention and ORP distribution in CWs[14,31]. VSSF CWs is usually considered to be a highly aerobic system while HSSF CWs is deemed as an anoxic system due to their oxygen transfer ability[31]. Thus, with the mixing effect of wastewater, ORP gradient in vertical orientation was usually negligible in VSSF CWs. However, higher ORP level in upper layer and lower level in bottom layer was frequently observed in HSSF CWs[21]. Nevertheless, higher hydraulic loading meant stronger mixing effect. Under high hydraulic loading condition, ORP gradient in vertical orientation of HSSF CWs did not always exist[20]. It could explain why VSSF CWs with low hydraulic loading showed a less spatial variation of TP retention than that done by HSSF CWs, and why the difference of TP spatial distribution in HSSF CWs was reduced when inflow loading was increased.

Furthermore, when treating wastewater consisted of high organic matter or nitrogen concentration, oxygen transfer could be significantly affected by pollutant distribution and microbial activity, which in return caused the redistribution of pH and ORP within these two types of system. In usual case, substrates in inflow section of constructed wetland system would first contact wastewater with higher phosphorus concentration than it did in outflow section[32], which was conducive to the adsorption of phosphorus. For the reason, higher phosphorus content in substrate was always found in inflow section. However, higher organic matters would consume more oxygen and cause lower redox condition in CWs[33]. On the other hand, higher hydraulic loading also meant shorter retention time and sometimes lower adsorption efficiency[34]. As a result, sharp reduction of TP content was observed in VSSF CWs when inflow loading was increased (Fig 2B). Moreover, non-rihzosphere substrates were faraway from the root system, where oxygen was always limited in poor oxygen transfer system. Thus, massive organic matter degradations(especially under high hydraulic loading condition) may accelerate the anaerobic hydrolysis of organic matter in this area, causing the decrease of pH level[17], reduction of Fe(Ⅲ) to Fe^2+^ [13,35], and extraction and release of Fe and Al [18], which eventually reduced the TP retention capacity [36]. As shown in Fig 2A, TP content in non-rihzosphere substrate in outflow section of HSSF CWs with low inflow loading was much lower than the control value, and TP content in non-rhizosphere substrate in inflow section was quite closed to the control value when hydraulic loading was increased.

Though the spatial variation of ORP and pH within the four CWs was not measured in this work, significant spatial distribution of inorganic P both in VSSF CWs and HSSF CWs had been observed (Fig 3–Fig 5), from which a relevant result could be inferred. Generally, the variation of Ca-P content in substrate was found to be correlated to the distribution of pH level. It was reported that when pH > 6, phosphorus could be adsorbed with iron and aluminum oxides and precipitated with calcium, while at lower pH levels, precipitation like Fe-P and Al-P, would become more dominant [37]. Thus, it seemed that high pH levels existed in those areas with high Ca-P content and vice versa. Besides pH level, oxidation–reduction condition was another key factor that affected the retention of Fe-P and Al-P in substrate. Therefore, the variation of Fe-P and Al-P could indirectly reflect the redox status of the substrate.

Fig 4 and Fig 5 revealed that distribution of Fe-P and Al-P content in substrates displayed a similar tendency of "rhizosphere">"near-rhizosphere"> "non-rhizosphere", which conformed to the transfer fate of oxygen from plant root to rhizosphere, then near-rhizonsphere and non-rhizosphere. On one hand, high Fe-P and Al-P content in rhizosphere substrates in all CWs indicated an aerobic condition in these areas. On the other hand, Fe-P release occurring in non-rhizosphere substrates in all HSSF CWs and in VSSF CWs with high hydraulic loading, signified an anaerobic redox situation of these substrates [38].

In addition, spatial distribution of Ca-P in HSSF CWs (Fig 3A) implied that pH level in rhizosphere substrate should be much higher than that in near-rhizosphere and non-rhizosphere in this type of CWs. However, adverse situation was observed in VSSF CWs (Fig 3B), which implied that pH level in non-rhizosphere substrate was at higher level than that in rhizosphere substrate.

### Relationship between Inorganic Phosphorus Distribution and TP Distribution

Correlation of TP with Ca-P, Fe-P and Al-P in four CWs was shown in Table 3. It could be seen that in HSSF CWs, spatial distribution of Ca-P had high correlation with distribution of Fe-P, Al-P and TP, however, in VSSF CWs, the correlation coefficients were much lower and even negative. The result implied that variation of pH level was positively correlated to the change of redox status in HSSF CWs, but negatively correlated to the change of redox status in VSSF CWs.

**Table 3:** Correlation between Ca-P, Fe-P, Al-P and TP in four CWs.

	Fe-P	Al-P	TP		Fe-P	Al-P	TP
VSSF(100*10^3^m/d)				HSSF(100*10^3^m/d)			
Ca-P	-0.404	-0.481	0.129	Ca-P	0.780**	0.823**	0.741**
Fe-P		0.939**	0.746**	Fe-P		0.898**	0.762**
Al-P			0.765**	Al-P			0.925**
VSSF(60*10^3^m/d)				HSSF(60*10^3^m/d)			
Ca-P	-0.610*	-0.461	-0.116	Ca-P	0.728**	0.577*	0.742**
Fe-P		0.874**	0.616*	Fe-P		0.926**	0.982**
Al-P			0.588*	Al-P			0.922**

* Correlation is significant at the 0.05 level (2-tailed).

** Correlation is significant at the 0.01 level (2-tailed).

As shown in Table 2, High COD removal rates were observed in both VSSF CWs and HSSF CWs in this work. However, removal rate of ammonium in VSSF CWs was much higher than that in HSSF CWs, indicating that nitrification process was enhanced in VSSF CWs. The results confirmed that VSSF CWs contained more oxygen than HSSF CWs in this work, and also suggested that most COD was removed under aerobic condition in VSSF CWs, and under anoxic or anaerobic condition in HSSF CWs. It was reported that pH values would decrease in those areas with excessive oxygen or insufficient oxygen due to nitrification or hydrolysis acidification[39,40]. In contrast, pH would maintain stable under moderately aerobic condition or slightly increase under denitrification condition.

In another word, the variation of redox status and pH level would be positively correlated under moderately aerobic condition and hydrolysis acidification condition, while was negatively correlated under nitrification or denitrification condition. Rhizosphere substrate and non-rhizosphere substrate in HSSF CWs with low inflow loading were the instances of the former situation, while rhizosphere substrate and non-rhizosphere substrate in VSSF CWs with low inflow loading were the examples of the latter situation. As a result, the highest P content was always found in the substrate with high pH and ORP level, while the lowest one was discovered in substrate with low pH and ORP level, and mediate values would be observed in low pH with high ORP or high pH with low ORP. Thus, substrates in HSSF CWs showed a more remarkable difference in TP and inorganic P spatial distribution than those in VSSF CWs in this work.

In addition, when inflow loading was increased, the situation of redox status and pH level variation would be reinforced due to the enhancement of microbial activity or a stronger mixing effect. With VSSF CWs, both the decrease of ORP and pH level in inflow section caused the sharp decrease of total TP content. In contrast, with HSSF CWs, ORP and pH level in inflow section decreased because of more oxygen consumption. However, the values increased due to the stronger oxygen transfer in outflow section, thus no significant influence on total TP retention of the whole system was observed.

### Effect of P Spatial Distribution on CWs Design and Operation

In this work, phosphorus was removed mainly through adsorption and precipitation by formation of Ca-P, Fe-P and Al-P. Thus, for phosphorus-rich wastewater treatment, P retention capacity was an important parameter for CWs design and operation, and spatial distribution of phosphorus should be taken into account while considering processes such as hydraulic loading design, substrate selection, longevity assessment and so on.

For instance, Fig 2 showed that variation of TP spatial distribution was reduced in VSSF CWs with low inflow loading and in HSSF CWs with high inflow loading. Therefore, for CWs hydraulic loading design, it seemed that low hydraulic loading was more suitable for VSSF CWs while high hydraulic loading was more appropriate for HSSF CWs.

Furthermore, Fig 2 also displayed that TP content in rhizonsphere substrates was much higher than that in non-rhizosphere substrate in HSSF CWs, which indicated that substrates in non-rhizonsphere may have a shorter longevity than those in rhizonsphere. Thus, for longevity assessment of certain type of CWs, samples in all areas should be taken and comprehensively evaluated to avoid misestimate due to the variety of TP retentions of substrate.

In addition, to improve the P retention capacity of CWs, filter medium with excellent adsorption capacity should be considered. In this respect, pattern of P spatial distribution provided a guideline for substrate construction. Generally, substrates with high P adsorption capacity had been wildly studied and could be classified into three catagories[41,9], calcium-rich medium like zonolite, iron-rich medium like blast furnace slags, and aluminum-rich medium like red mud. Therefore, the substrate selection could be conducted according to distribution of Ca-P, Fe-P and Al-P. For example, iron-rich and aluminum-rich mediums were more efficient than calcium-rich medium when used as rihzoshpere substrates in VSSF CWs with low inflow loading, since Ca-P retention capacity in these areas was lower than Fe-P and Al-P. In contrast, calcium-rich medium should be considered first in non-rhizosphere substrates in HSSF CWs with high inflow loading because the retention capacity of Fe-P and Al-P were rather low in these areas.

## Conclusions

After 5 months of operation for synthetic wastewater treatment, significant spatial variations of TP and inorganic phosphorus(Ca-P, Fe-P and Al-P) in substrates were observed, which were greatly affected by types of CWs and hydraulic loading. Though the spatial distribution of Fe-P and Al-P showed a similar order of content in substrate as "rhizosphere" > "near-rhizosphere" > "non-rhizosphere" regardless of CWs type and inflow loading, spatial distribution of Ca-P was positively correlated to that of Fe-P and Al-P in HSSF CWs, while it was negatively correlated to that of Fe-P and Al-P in VSSF CWs. As a result, TP spatial distribution in HSSF CWs showed a greater diversity than that in VSSF CWs. However, TP spatial difference decreased when hydraulic loading was increased in both types of CWs. These results suggested that P spatial distribution should be taken into account for CWs design and operation.

## Supporting Information

S1 Table Original data for Fig 2A and Fig 2B in this manuscript. (DOC)

S2 Table Original data for Fig 3A and Fig 3B in this manuscript. (DOC)

S3 Table Original data for Fig 4A and Fig 4B in this manuscript. (DOC)

S4 Table Original data for Fig 5A and Fig 5B in this manuscript. (DOC)

## References

[1] Liu C, Xu K, Inamori R, Ebie Y, Liao J, Inamori Y (2009) Pilot-scale studies of domestic wastewater treatment by typical constructed wetlands and their greenhouse gas emissions. Frontiers of Environmental Science & Engineering in China 3: 477–482.

[2] Bryhn AC (2009) Sustainable Phosphorus Loadings from Effective and Cost-Effective Phosphorus Management Around the Baltic Sea. PLoS ONE 4(5): e5417 10.1371/journal.pone.0005417 19412551 PMC2673029

[3] O’Neill A, Foy R, Phillips D (2011) Phosphorus retention in a constructed wetland system used to treat dairy wastewater. Bioresource technology 102: 5024–5031. 10.1016/j.biortech.2011.01.075 21367602

[4] Yang Y, Wang Z, Liu C, Guo X (2012) Enhanced P, N and C removal from domestic wastewater using constructed wetland employing construction solid waste (CSW) as main substrate. Water Science & Technology 66: 1022–1028. 22797230 10.2166/wst.2012.277

[5] Reddy K, D'angelo E (1997) Biogeochemical indicators to evaluate pollutant removal efficiency in constructed wetlands. Water Science and Technology 35: 1–10.

[6] Vymazal J (2007) Removal of nutrients in various types of constructed wetlands. Science of the Total Environment 380: 48–65. 17078997 10.1016/j.scitotenv.2006.09.014

[7] Drizo A, Comeau Y, Forget C, Chapuis RP (2002) Phosphorus saturation potential: a parameter for estimating the longevity of constructed wetland systems. Environmental Science & Technology 36: 4642–4648. 12433176 10.1021/es011502v

[8] Johansson Westholm L (2006) Substrates for phosphorus removal—Potential benefits for on-site wastewater treatment? Water Research 40: 23–36. 16375946 10.1016/j.watres.2005.11.006

[9] Vohla C, Kõiv M, Bavor HJ, Chazarenc F, Mander Ü (2011) Filter materials for phosphorus removal from wastewater in treatment wetlands—A review. Ecological Engineering 37: 70–89.

[10] Golterman H (1995) The labyrinth of nutrient cycles and buffers in wetlands: results based on research in the Camargue (southern France). Hydrobiologia 315: 39–58.

[11] Yates CR, Prasher SO (2009) Phosphorus reduction from agricultural runoff in a pilot-scale surface-flow constructed wetland. Ecological Engineering 35: 1693–1701.

[12] Moustafa M, White J, Coghlan C, Reddy K (2012) Influence of hydropattern and vegetation on phosphorus reduction in a constructed wetland under high and low mass loading rates. Ecological Engineering 42: 134–145.

[13] Reddy K, Kadlec R, Flaig E, Gale P (1999) Phosphorus retention in streams and wetlands: a review. Critical reviews in environmental science and technology 29: 83–146.

[14] Faulwetter JL, Gagnon V, Sundberg C, Chazarenc F, Burr MD, Brisson J, et al (2009) Microbial processes influencing performance of treatment wetlands: a review. Ecological Engineering 35: 987–1004.

[15] White J, Reddy K, Majer-Newman J (2006) Hydrologic and vegetation effects on water column phosphorus in wetland mesocosms. Soil Science Society of America Journal 70: 1242–1251.

[16] White JR, Ramesh Reddy K, Moustafa M (2004) Influence of hydrologic regime and vegetation on phosphorus retention in Everglades stormwater treatment area wetlands. Hydrological processes 18: 343–355.

[17] Erler DV, Tait D, Eyre BD, Bingham M (2011) Observations of nitrogen and phosphorus biogeochemistry in a surface flow constructed wetland. Science of the Total Environment 409: 5359–5367. 10.1016/j.scitotenv.2011.08.052 21959246

[18] Babatunde A, Zhao Y (2009) Forms, patterns and extractability of phosphorus retained in alum sludge used as substrate in laboratory-scale constructed wetland systems. Chemical Engineering Journal 152: 8–13.

[19] García J, Ojeda E, Sales E, Chico F, Píriz T, Aguirre P, et al (2003) Spatial variations of temperature, redox potential, and contaminants in horizontal flow reed beds. Ecological Engineering 21: 129–142.

[20] Headley TR, Herity E, Davison L (2005) Treatment at different depths and vertical mixing within a 1-m deep horizontal subsurface-flow wetland. Ecological Engineering 25: 567–582.

[21] Ding Y, Wang W, Song X-s, Wang Y-h (2014) Spatial distribution characteristics of environmental parameters and nitrogenous compounds in horizontal subsurface flow constructed wetland treating high nitrogen-content wastewater. Ecological Engineering 70: 446–449.

[22] Tietz A, Kirschner A, Langergraber G, Sleytr K, Haberl R (2007) Characterisation of microbial biocoenosis in vertical subsurface flow constructed wetlands. Science of the Total Environment 380: 163–172. 17223185 10.1016/j.scitotenv.2006.11.034

[23] Truu M, Juhanson J, Truu J (2009) Microbial biomass, activity and community composition in constructed wetlands. Science of the Total Environment 407: 3958–3971. 10.1016/j.scitotenv.2008.11.036 19157517

[24] Seo DC, Cho JS, Lee HJ, Heo JS (2005) Phosphorus retention capacity of filter media for estimating the longevity of constructed wetland. Water Research 39: 2445–2457. 15978654 10.1016/j.watres.2005.04.032

[25] Martín M, Gargallo S, Hernández-Crespo C, Oliver N (2013) Phosphorus and nitrogen removal from tertiary treated urban wastewaters by a vertical flow constructed wetland. Ecological Engineering 61: 34–42.

[26] Bezbaruah AN, Zhang TC (2004) pH, redox, and oxygen microprofiles in rhizosphere of bulrush (*Scirpus validus*) in a constructed wetland treating municipal wastewater. Biotechnology and bioengineering 88: 60–70. 15384055 10.1002/bit.20208

[27] Cao XY, Chong YX, Yu GW, Zhong HT (2012) Difference of P Content in Different Area Substrate of Constructed Wetland, Environmental Science 33, 4033–4039. 23323442

[28] Bao SD (2000) Soil and agricultural chemistry analysis Chinese Agric Press, Beijing.

[29] SEPA of PRC (2002)Technical Specifications Requirements for Monitoring of Surface Water and Waste Water.

[30] Li L, Li Y, Biswas DK, Nian Y, Jiang G (2008) Potential of constructed wetlands in treating the eutrophic water: evidence from Taihu Lake of China. Bioresource technology 99: 1656–1663. 17532209 10.1016/j.biortech.2007.04.001

[31] Herrmann I, Jourak A, Hedström A, Lundström TS, Viklander M (2013) The Effect of Hydraulic Loading Rate and Influent Source on the Binding Capacity of Phosphorus Filters. PLoS ONE 8(8): e69017 10.1371/journal.pone.0069017 23936313 PMC3732246

[32] Maine M, Sune N, Hadad H, Sánchez G (2007) Temporal and spatial variation of phosphate distribution in the sediment of a free water surface constructed wetland. Science of the Total Environment 380: 75–83. 17229453 10.1016/j.scitotenv.2006.11.036

[33] Richardson J, Vepraskas M (2000) Wetland soils: Their genesis, hydrology, landscape, and separation into hydric and nonhydric soils Ann Arbor, Mich: Ann Arbor Science Publishers.

[34] Trang NTD, Konnerup D, Schierup H-H, Chiem NH, Tuan LA, Brix H. (2010) Kinetics of pollutant removal from domestic wastewater in a tropical horizontal subsurface flow constructed wetland system: effects of hydraulic loading rate. Ecological Engineering 36: 527–535.

[35] Chen M, Ye T-R, Krumholz LR, Jiang H-L (2014) Temperature and Cyanobacterial Bloom Biomass Influence Phosphorous Cycling in Eutrophic Lake Sediments. PLoS ONE 9(3): e93130 10.1371/journal.pone.0093130 24682039 PMC3969358

[36] van Diggelen JMH, Lamers LPM, van Dijk G, Schaafsma MJ, Roelofs JGM, Smolders AJP (2014) New Insights into Phosphorus Mobilisation from Sulphur-Rich Sediments: Time-Dependent Effects of Salinisation. PLoS ONE 9(11): e111106 10.1371/journal.pone.0111106 25369128 PMC4219700

[37] Arias C, Del Bubba M, Brix H (2001) Phosphorus removal by sands for use as media in subsurface flow constructed reed beds. Water Research 35: 1159–1168. 11268836 10.1016/s0043-1354(00)00368-7

[38] Ann Y, Reddy K, Delfino J (1999) Influence of chemical amendments on phosphorus immobilization in soils from a constructed wetland. Ecological Engineering 14: 157–167.

[39] Mnch C, Kuschk P, Rske I (2005) Root stimulated nitrogen removal: only a local effect or important for water treatment? Water Science & Technology 51: 185–192. 16042258

[40] Dickopp J, Kazda M, Čížková H (2011) Differences in rhizome aeration of *Phragmites australis* in a constructed wetland. Ecological Engineering 37: 1647–1653.

[41] Drizo A, Frost C, Grace J, Smith K (1999) Physico-chemical screening of phosphate-removing substrates for use in constructed wetland systems. Water Research 33: 3595–3602.

