# Correction: Spatial Variation of Phosphorous Retention Capacity in Subsurface Flow Constructed Wetlands: Effect of Wetland Type and Inflow Loading

**DOI:** 10.1371/journal.pone.0149705

**Published:** 2016-02-12

**Authors:** Guangwei Yu, Meijuan Tan, Yunxiao Chong, Xinxian Long

There is an error in the Funding section. The correct funding information is as follows: The study was funded by National Natural Science Foundation of China (No. 51378226). It had an important role in study design, data collection and analysis.
