# Supplementary material for: Spatial Variation of Phosphorous Retention Capacity in Subsurface Flow Constructed Wetlands: Effect of Wetland Type and Inflow Loading
Source: PLoS One. 2015 Jul 28;10(7):e0134010. doi: 10.1371/journal.pone.0134010 (PMC4517764; doi:10.1371/journal.pone.0134010)
Supplement: S1 Table — (DOC) [file pone.0134010.s001.doc]

Table 1. Data for Fig 2 a.

| HSSF CWs |  |  |  |  |  |  |  |  |  |
| --- | --- | --- | --- | --- | --- | --- | --- | --- | --- |
| hydraulic load：100*10^3^ m/d | | | | | hydraulic load：60*10^3^ m/d | | | | |
|  |  | TP（g/kg） | | |  |  | TP（g/kg） | | |
|  | Samples |  | Mean | SD |  | Samples |  | Mean | SD |
| rhizosphere (inflow) | 1 | 2.15 |  |  | rhizosphere (inflow) | 1 | 1.81 |  |  |
|  | 2 | 2.22 |  |  |  | 2 | 2.64 |  |  |
|  | 3 | 2.34 | 2.24 | 0.09 |  | 3 | 2.72 | 2.39 | 0.50 |
| near-rhizosphere (inflow) | 1 | 0.48 |  |  | near-rhizosphere (inflow) | 1 | 1.89 |  |  |
|  | 2 | 0.45 |  |  |  | 2 | 1.89 |  |  |
|  | 3 | 0.42 | 0.45 | 0.03 |  | 3 | 1.81 | 1.86 | 0.04 |
| non-rhizosphere (inflow) | 1 | 0.26 |  |  | non-rhizosphere (inflow) | 1 | 0.85 |  |  |
|  | 2 | 0.24 |  |  |  | 2 | 0.94 |  |  |
|  | 3 | 0.27 | 0.26 | 0.02 |  | 3 | 1.02 | 0.93 | 0.08 |
| rhizosphere (outflow) | 1 | 2.19 |  |  | rhizosphere (outflow) | 1 | 1.32 |  |  |
|  | 2 | 2.22 |  |  |  | 2 | 1.34 |  |  |
|  | 3 | 2.06 | 2.15 | 0.09 |  | 3 | 1.29 | 1.32 | 0.02 |
| near-rhizosphere (outflow) | 1 | 0.33 |  |  | near-rhizosphere (outflow) | 1 | 0.13 |  |  |
|  | 2 | 0.43 |  |  |  | 2 | 0.17 |  |  |
|  | 3 | 0.36 | 0.38 | 0.05 |  | 3 | 0.11 | 0.14 | 0.03 |
| non-rhizosphere (outflow) | 1 | 0.59 |  |  | non-rhizosphere (outflow) | 1 | 0.15 |  |  |
|  | 2 | 0.60 |  |  |  | 2 | 0.15 |  |  |
|  | 3 | 0.63 | 0.60 | 0.02 |  | 3 | 0.17 | 0.16 | 0.01 |
| Control value | 1 | 0.1967 |  |  |  |  |  |  |  |
|  | 2 | 0.2368 |  |  |  |  |  |  |  |
|  | 3 | 0.1876 | 0.21 | 0.03 |  |  |  |  |  |

Table 2. Data for Fig 2 b.

| VSSF CWs |  |  |  |  |  |  |  |  |  |
| --- | --- | --- | --- | --- | --- | --- | --- | --- | --- |
| hydraulic load：100*10^3^ m/d | |  |  |  | hydraulic load：60*10^3^ m/d | |  |  |  |
|  |  | TP（g/kg） | | |  |  | TP（g/kg） | | |
|  | Samples |  | Mean | SD |  | Samples |  | Mean | SD |
| rhizosphere | 1 | 0.58 |  |  | rhizosphere | 1 | 1.13 |  |  |
|  | 2 | 0.66 |  |  |  | 2 | 1.01 |  |  |
|  | 3 | 0.66 | 0.63 | 0.05 |  | 3 | 1.03 | 1.06 | 0.06 |
| near-rhizosphere | 1 | 0.29 |  |  | near-rhizosphere | 1 | 1.05 |  |  |
|  | 2 | 0.22 |  |  |  | 2 | 1.05 |  |  |
|  | 3 | 0.29 | 0.26 | 0.04 |  | 3 | 1.14 | 1.08 | 0.05 |
| non-rhizosphere (inflow) | 1 | 0.18 |  |  | non-rhizosphere (inflow) | 1 | 0.97 |  |  |
|  | 2 | 0.20 |  |  |  | 2 | 1.03 |  |  |
|  | 3 | 0.25 | 0.21 | 0.03 |  | 3 | 1.00 | 1.00 | 0.03 |
| non-rhizosphere (outflow) | 1 | 0.34 |  |  | non-rhizosphere (outflow) | 1 | 0.76 |  |  |
|  | 2 | 0.39 |  |  |  | 2 | 0.76 |  |  |
|  | 3 | 0.27 | 0.33 | 0.06 |  | 3 | 0.80 | 0.78 | 0.02 |
| Control value | 1 | 0.1967 |  |  |  |  |  |  |  |
|  | 2 | 0.2368 |  |  |  |  |  |  |  |
|  | 3 | 0.1876 | 0.21 | 0.03 |  |  |  |  |  |
