# Supplementary material for: Spatial Variation of Phosphorous Retention Capacity in Subsurface Flow Constructed Wetlands: Effect of Wetland Type and Inflow Loading
Source: PLoS One. 2015 Jul 28;10(7):e0134010. doi: 10.1371/journal.pone.0134010 (PMC4517764; doi:10.1371/journal.pone.0134010)
Supplement: S2 Table — (DOC) [file pone.0134010.s002.doc]

Table 1. Data for Fig. 3 a.

| HSSF CWs |  |  |  |  |  |  |  |  |  |
| --- | --- | --- | --- | --- | --- | --- | --- | --- | --- |
| hydraulic load：100*10^3^ m/d | | | | | hydraulic load：60*10^3^ m/d | | | | |
|  |  | Ca-P（mg/kg） | | |  |  | Ca-P（mg/kg） | | |
|  | Samples |  | Mean | SD |  | Samples |  | Mean | SD |
| rhizosphere (inflow) | 1 | 367.78 |  |  | rhizosphere (inflow) | 1 | 224.37 |  |  |
|  | 2 | 192.51 |  |  |  | 2 | 387.21 |  |  |
|  | 3 | 335.51 | 298.60 | 93.28 |  | 3 | 406.09 | 339.22 | 99.91 |
| near-rhizosphere (inflow) | 1 | 114.86 |  |  | near-rhizosphere (inflow) | 1 | 130.02 |  |  |
|  | 2 | 103.19 |  |  |  | 2 | 95.06 |  |  |
|  | 3 | 133.80 | 117.28 | 15.45 |  | 3 | 185.00 | 136.69 | 45.34 |
| non-rhizosphere (inflow) | 1 | 105.01 |  |  | non-rhizosphere (inflow) | 1 | 154.06 |  |  |
|  | 2 | 100.34 |  |  |  | 2 | 170.15 |  |  |
|  | 3 | 103.54 | 102.96 | 2.39 |  | 3 | 116.92 | 147.04 | 27.30 |
| rhizosphere (outflow) | 1 | 198.23 |  |  | rhizosphere (outflow) | 1 | 301.08 |  |  |
|  | 2 | 153.95 |  |  |  | 2 | 330.65 |  |  |
|  | 3 | 109.60 | 153.93 | 44.31 |  | 3 | 271.03 | 300.92 | 29.81 |
| near-rhizosphere (outflow) | 1 | 92.58 |  |  | near-rhizosphere (outflow) | 1 | 90.16 |  |  |
|  | 2 | 92.83 |  |  |  | 2 | 90.35 |  |  |
|  | 3 | 70.71 | 85.37 | 12.70 |  | 3 | 107.51 | 96.01 | 9.96 |
| non-rhizosphere (outflow) | 1 | 83.62 |  |  | non-rhizosphere (outflow) | 1 | 56.40 |  |  |
|  | 2 | 75.91 |  |  |  | 2 | 58.40 |  |  |
|  | 3 | 117.28 | 92.27 | 22.00 |  | 3 | 58.69 | 57.83 | 1.25 |
| Control value | 1 | 38.76 |  |  |  |  |  |  |  |
|  | 2 | 58.19 |  |  |  |  |  |  |  |
|  | 3 | 61.07 | 52.67 | 12.14 |  |  |  |  |  |

Table 2. Data for Fig. 3 b.

| VSSF CWs |  |  |  |  |  |  |  |  |  |
| --- | --- | --- | --- | --- | --- | --- | --- | --- | --- |
| hydraulic load：100*10^3^ m/d | |  |  |  | hydraulic load：60*10^3^ m/d | |  |  |  |
|  |  | Ca-P（mg/kg） | | |  |  | Ca-P（mg/kg） | | |
|  | Samples |  | Mean | SD |  | Samples |  | Mean | SD |
| rhizosphere | 1 | 105.48 |  |  | rhizosphere | 1 | 212.62 |  |  |
|  | 2 | 198.25 |  |  |  | 2 | 197.74 |  |  |
|  | 3 | 78.27 | 127.33 | 62.90 |  | 3 | 185.66 | 198.67 | 13.50 |
| near-rhizosphere | 1 | 95.37 |  |  | near-rhizosphere | 1 | 295.68 |  |  |
|  | 2 | 71.04 |  |  |  | 2 | 303.68 |  |  |
|  | 3 | 58.70 | 75.03 | 18.66 |  | 3 | 313.47 | 304.28 | 8.91 |
| non-rhizosphere (inflow) | 1 | 88.08 |  |  | non-rhizosphere (inflow) | 1 | 481.78 |  |  |
|  | 2 | 90.54 |  |  |  | 2 | 248.90 |  |  |
|  | 3 | 78.47 | 85.70 | 6.38 |  | 3 | 370.28 | 366.99 | 116.47 |
| non-rhizosphere (outflow) | 1 | 303.58 |  |  | non-rhizosphere (outflow) | 1 | 225.42 |  |  |
|  | 2 | 247.45 |  |  |  | 2 | 314.57 |  |  |
|  | 3 | 269.44 | 273.49 | 28.29 |  | 3 | 321.52 | 287.17 | 53.59 |
| Control value | 1 | 38.76 |  |  |  |  |  |  |  |
|  | 2 | 58.19 |  |  |  |  |  |  |  |
|  | 3 | 61.07 | 52.67 | 12.14 |  |  |  |  |  |
