# Supplementary material for: Spatial Variation of Phosphorous Retention Capacity in Subsurface Flow Constructed Wetlands: Effect of Wetland Type and Inflow Loading
Source: PLoS One. 2015 Jul 28;10(7):e0134010. doi: 10.1371/journal.pone.0134010 (PMC4517764; doi:10.1371/journal.pone.0134010)
Supplement: S3 Table — (DOC) [file pone.0134010.s003.doc]

Table 1. Data for Fig. 4 a.

| HSSF CWs |  |  |  |  |  |  |  |  |  |
| --- | --- | --- | --- | --- | --- | --- | --- | --- | --- |
| hydraulic load：100*10^3^ m/d | | | | | hydraulic load：60*10^3^ m/d | | | | |
|  |  | Fe-P（mg/kg） | | |  |  | Fe-P（mg/kg） | | |
|  | Samples |  | Mean | SD |  | Samples |  | Mean | SD |
| rhizosphere (inflow) | 1 | 202.16 |  |  | rhizosphere (inflow) | 1 | 244.85 |  |  |
|  | 2 | 204.69 |  |  |  | 2 | 282.32 |  |  |
|  | 3 | 185.14 | 197.33 | 10.63 |  | 3 | 233.38 | 253.52 | 25.60 |
| near-rhizosphere (inflow) | 1 | 79.18 |  |  | near-rhizosphere (inflow) | 1 | 178.59 |  |  |
|  | 2 | 78.62 |  |  |  | 2 | 186.72 |  |  |
|  | 3 | 72.01 | 76.60 | 3.99 |  | 3 | 184.51 | 183.27 | 4.20 |
| non-rhizosphere (inflow) | 1 | 2.44 |  |  | non-rhizosphere (inflow) | 1 | 60.16 |  |  |
|  | 2 | 2.94 |  |  |  | 2 | 48.83 |  |  |
|  | 3 | 2.47 | 2.61 | 0.28 |  | 3 | 47.26 | 52.08 | 7.04 |
| rhizosphere (outflow) | 1 | 106.21 |  |  | rhizosphere (outflow) | 1 | 132.18 |  |  |
|  | 2 | 99.21 |  |  |  | 2 | 127.36 |  |  |
|  | 3 | 89.14 | 98.19 | 8.58 |  | 3 | 112.81 | 124.12 | 10.09 |
| near-rhizosphere (outflow) | 1 | 86.73 |  |  | near-rhizosphere (outflow) | 1 | 6.82 |  |  |
|  | 2 | 85.50 |  |  |  | 2 | 8.30 |  |  |
|  | 3 | 84.85 | 85.70 | 0.96 |  | 3 | 7.33 | 7.49 | 0.75 |
| non-rhizosphere (outflow) | 1 | 11.31 |  |  | non-rhizosphere (outflow) | 1 | 13.24 |  |  |
|  | 2 | 10.77 |  |  |  | 2 | 13.63 |  |  |
|  | 3 | 10.75 | 10.95 | 0.32 |  | 3 | 13.20 | 13.36 | 0.23 |
| Control value | 1 | 19.60 |  |  |  |  |  |  |  |
|  | 2 | 23.49 |  |  |  |  |  |  |  |
|  | 3 | 22.02 | 21.70 | 1.97 |  |  |  |  |  |

Table 2. Data for Fig. 4 b.

| VSSF CWs |  |  |  |  |  |  |  |  |  |
| --- | --- | --- | --- | --- | --- | --- | --- | --- | --- |
| hydraulic load：100*10^3^ m/d | |  |  |  | hydraulic load：60*10^3^ m/d | |  |  |  |
|  |  | Fe-P（mg/kg） | | |  |  | Fe-P（mg/kg） | | |
|  | Samples |  | Mean | SD |  | Samples |  | Mean | SD |
| rhizosphere | 1 | 155.51 |  |  | rhizosphere | 1 | 204.31 |  |  |
|  | 2 | 143.43 |  |  |  | 2 | 160.63 |  |  |
|  | 3 | 137.47 | 145.47 | 9.20 |  | 3 | 208.62 | 191.19 | 26.55 |
| near-rhizosphere | 1 | 88.03 |  |  | near-rhizosphere | 1 | 79.66 |  |  |
|  | 2 | 104.35 |  |  |  | 2 | 102.37 |  |  |
|  | 3 | 63.10 | 85.16 | 20.77 |  | 3 | 87.18 | 89.74 | 11.57 |
| non-rhizosphere (inflow) | 1 | 24.95 |  |  | non-rhizosphere (inflow) | 1 | 35.71 |  |  |
|  | 2 | 30.34 |  |  |  | 2 | 42.95 |  |  |
|  | 3 | 39.73 | 31.67 | 7.47 |  | 3 | 41.69 | 40.11 | 3.87 |
| non-rhizosphere (outflow) | 1 | 14.20 |  |  | non-rhizosphere (outflow) | 1 | 16.17 |  |  |
|  | 2 | 13.72 |  |  |  | 2 | 33.65 |  |  |
|  | 3 | 17.15 | 15.02 | 1.86 |  | 3 | 28.47 | 26.10 | 8.98 |
| Control value | 1 | 19.60 |  |  |  |  |  |  |  |
|  | 2 | 23.49 |  |  |  |  |  |  |  |
|  | 3 | 22.02 | 21.70 | 1.97 |  |  |  |  |  |
