# Supplementary material for: Spatial Variation of Phosphorous Retention Capacity in Subsurface Flow Constructed Wetlands: Effect of Wetland Type and Inflow Loading
Source: PLoS One. 2015 Jul 28;10(7):e0134010. doi: 10.1371/journal.pone.0134010 (PMC4517764; doi:10.1371/journal.pone.0134010)
Supplement: S4 Table — (DOC) [file pone.0134010.s004.doc]

Table 1. Data for Fig. 5 a.

| HSSF CWs |  |  |  |  |  |  |  |  |  |
| --- | --- | --- | --- | --- | --- | --- | --- | --- | --- |
| hydraulic load：100*10^3^ m/d | | | | | hydraulic load：60*10^3^ m/d | | | | |
|  |  | Al-P（mg/kg） | | |  |  | Al-P（mg/kg） | | |
|  | Samples |  | Mean | SD |  | Samples |  | Mean | SD |
| rhizosphere (inflow) | 1 | 161.48 |  |  | rhizosphere (inflow) | 1 | 161.48 |  |  |
|  | 2 | 166.19 |  |  |  | 2 | 166.19 |  |  |
|  | 3 | 164.82 | 164.16 | 2.42 |  | 3 | 164.82 | 164.16 | 2.42 |
| near-rhizosphere (inflow) | 1 | 37.88 |  |  | near-rhizosphere (inflow) | 1 | 37.88 |  |  |
|  | 2 | 46.19 |  |  |  | 2 | 46.19 |  |  |
|  | 3 | 87.58 | 57.22 | 26.62 |  | 3 | 87.58 | 57.22 | 26.62 |
| non-rhizosphere (inflow) | 1 | 10.99 |  |  | non-rhizosphere (inflow) | 1 | 10.99 |  |  |
|  | 2 | 17.13 |  |  |  | 2 | 17.13 |  |  |
|  | 3 | 13.56 | 13.89 | 3.08 |  | 3 | 13.56 | 13.89 | 3.08 |
| rhizosphere (outflow) | 1 | 141.45 |  |  | rhizosphere (outflow) | 1 | 141.45 |  |  |
|  | 2 | 108.99 |  |  |  | 2 | 108.99 |  |  |
|  | 3 | 112.28 | 120.91 | 17.87 |  | 3 | 112.28 | 120.91 | 17.87 |
| near-rhizosphere (outflow) | 1 | 40.69 |  |  | near-rhizosphere (outflow) | 1 | 40.69 |  |  |
|  | 2 | 35.67 |  |  |  | 2 | 35.67 |  |  |
|  | 3 | 33.16 | 36.50 | 3.83 |  | 3 | 33.16 | 36.50 | 3.83 |
| non-rhizosphere (outflow) | 1 | 19.92 |  |  | non-rhizosphere (outflow) | 1 | 19.92 |  |  |
|  | 2 | 20.08 |  |  |  | 2 | 20.08 |  |  |
|  | 3 | 19.30 | 19.77 | 0.41 |  | 3 | 19.30 | 19.77 | 0.41 |
| Control value | 1 | 4.22 |  |  |  |  |  |  |  |
|  | 2 | 4.24 |  |  |  |  |  |  |  |
|  | 3 | 4.97 | 4.47 | 0.43 |  |  |  |  |  |

Table 2. Data for Fig. 5 b.

| VSSF CWs |  |  |  |  |  |  |  |  |  |
| --- | --- | --- | --- | --- | --- | --- | --- | --- | --- |
| hydraulic load：100*10^3^ m/d | |  |  |  | hydraulic load：60*10^3^ m/d | |  |  |  |
|  |  | Al-P（mg/kg） | | |  |  | Al-P（mg/kg） | | |
|  | Samples |  | Mean | SD |  | Samples |  | Mean | SD |
| rhizosphere | 1 | 76.04 |  |  | rhizosphere | 1 | 92.62 |  |  |
|  | 2 | 70.73 |  |  |  | 2 | 113.03 |  |  |
|  | 3 | 96.62 | 81.13 | 13.67 |  | 3 | 142.91 | 116.19 | 25.29 |
| near-rhizosphere | 1 | 52.82 |  |  | near-rhizosphere | 1 | 65.49 |  |  |
|  | 2 | 42.62 |  |  |  | 2 | 63.18 |  |  |
|  | 3 | 49.41 | 48.28 | 5.19 |  | 3 | 60.98 | 63.22 | 2.25 |
| non-rhizosphere (inflow) | 1 | 23.98 |  |  | non-rhizosphere (inflow) | 1 | 62.12 |  |  |
|  | 2 | 23.74 |  |  |  | 2 | 61.98 |  |  |
|  | 3 | 30.41 | 26.04 | 3.78 |  | 3 | 74.79 | 66.30 | 7.36 |
| non-rhizosphere (outflow) | 1 | 12.49 |  |  | non-rhizosphere (outflow) | 1 | 31.85 |  |  |
|  | 2 | 12.25 |  |  |  | 2 | 33.90 |  |  |
|  | 3 | 15.43 | 13.39 | 1.77 |  | 3 | 35.59 | 33.78 | 1.87 |
| Control value | 1 | 4.22 |  |  |  |  |  |  |  |
|  | 2 | 4.24 |  |  |  |  |  |  |  |
|  | 3 | 4.97 | 4.47 | 0.43 |  |  |  |  |  |
